# microRNA deregulation in Hutchinson-Gilford Progeria

**DOI:** 10.1186/1750-1172-10-S2-O15

**Published:** 2015-11-11

**Authors:** Patrice Roll

**Affiliations:** 1 Aix Marseille Université, INSERM, GMGF UMR_S 910 & Department of Medical Genetics and Cell Biology, La Timone Children's hospital, APHM, 13385, Marseille, France

## 

The Hutchinson-Gilford Progeria Syndrome (HGPS) is a rare genetic disease characterized by an accelerated aging, due to the accumulation in nucleus of a toxic protein called progerin, leading to abnormal gene expression and potential microRNA (miRNA) deregulation. To evaluate the role of miRNAs in HGPS, we conducted an *in vitro* miRNome analysis by RT-qPCR on dermal fibroblasts of 5 patients and 5 healthy individuals at early (P12+/-2) and for 5 individuals at late passages (P22+/-2). We found 29 deregulated miRNAs in more than 50% of patients (15 overexpressed, 14 underexpressed) presenting different deregulation profiles depending on their age and passage *in vitro*. We identified 4 interesting potential targeted pathways linked to aging/Progeria: cell cycle and proliferation, senescence, inflammation and autophagy for which 3 miRNAs target central actors of this pathway. No significant difference between patients and controls was detected for 3 autophagy makers on western blotting. However, using flow cytometry, allowing quantification of autophagy level cell by cell, we observed in a 14 yo patient exhibiting the most miRNA deregulated profile, a majority of cells presenting no autophagy. Our hypothesis is that the combined overexpression of the 3 autophagy inhibitor miRNAs acts as a “brake” on autophagy, leading to a decrease of progerin degradation, and finally to a pathophysiological vicious cycle. We are now confirming this hypothesis by transfecting antagomiRs on cellular model. We will also evaluate this mechanism in our HGPS LAKI mouse model and in the context of physiological aging, during which progerin is also produced at lower levels.

